# Evidence for divergent endocrine regulation of the murine and ovine GnRH receptor gene promoters

**DOI:** 10.3389/fendo.2025.1597028

**Published:** 2025-07-29

**Authors:** Christianne Magee, Jennifer E. Kouri, Brian D. Cherrington, Jeremy D. Cantlon, Dilyara A. Murtazina, Todd A. Farmerie, Meredith H. Davidsen, Terry M. Nett, Colin M. Clay

**Affiliations:** ^1^ Animal Reproduction and Biotechnology Laboratory, Department of Biomedical Sciences, Colorado State University, Fort Collins, CO, United States; ^2^ College of Agriculture, Life Sciences, and Natural Resources, Department of Zoology and Physiology, University of Wyoming, Laramie, WY, United States

**Keywords:** GnRH receptor, activin, estrogen gene expression, transgenic mice, follistatin

## Abstract

Activin, GnRH, and estrogen are key endocrine inputs known to regulate the GnRH receptor (*GnRHR*) promoter; however, it has become increasingly evident that the mechanisms regulating the *GnRHR* promoter vary by model and species. To explore these differences, transgenic mice harboring either a wild-type mouse *GnRHR* (*mGnRHR*) or sheep (*oGnRHR*) *GnRHR* promoter fused to luciferase (-LUC) were infected with an adenovirus overexpressing follistatin, neutralizing activin and decreasing serum concentrations of FSH in both animal models. However, follistatin overexpression in the oGnRHR-LUC mouse more than doubled luciferase expression, whereas in the mGnRHR-LUC animals it led to a 40% decrease in luciferase expression. Thus, the divergent transcriptional responses of the mouse and sheep *GnRHR* genes to activin appear to be reliably recapitulated in transgenic mice. To further elucidate mechanisms regulating *oGnRHR* expression, a mouse with a mutated cyclic AMP response element (µCRE) in the proximal oGnRHR-LUC promoter was developed. Using an electrophoretic mobility shift assay, a specific and high affinity interaction of the ovine CRE with nuclear components exists, but these are not modified in the presence of E_2,_ indicating that CRE binding protein (CREB) is necessary but not sufficient to mediate E_2_ input to *oGnRHR* expression.

## Introduction

Reproductive function is dependent on the binding of Gonadotropin Releasing Hormone (GnRH) to GnRH receptors (GnRHR) located on gonadotropes in the anterior pituitary gland, resulting in the subsequent synthesis and secretion of Luteinizing Hormone (LH) and Follicle Stimulating Hormone (FSH). As an activator of gonadotropin synthesis and secretion, the pulsatile release of GnRH also stimulates transcription of its cognate receptor in all species studied to date (1–5). While the dynamic pulsatile release of GnRH from the hypothalamus is significant (6–9), the relative abundance of GnRH receptors in the pituitary is equally important. More than 200 genes are regulated by agonist binding to the GnRHR, and the amplitude of expression for those genes is highly dependent on the number of GnRHR present at the cell membrane (10). Therefore, our efforts to illuminate the required cascade of events that culminates in normal reproductive function converge upon the coordination of expression of *GnRHR*, which we know to be regulated by three primary inputs: GnRH, activin, and estrogen.

**Figure 1:**
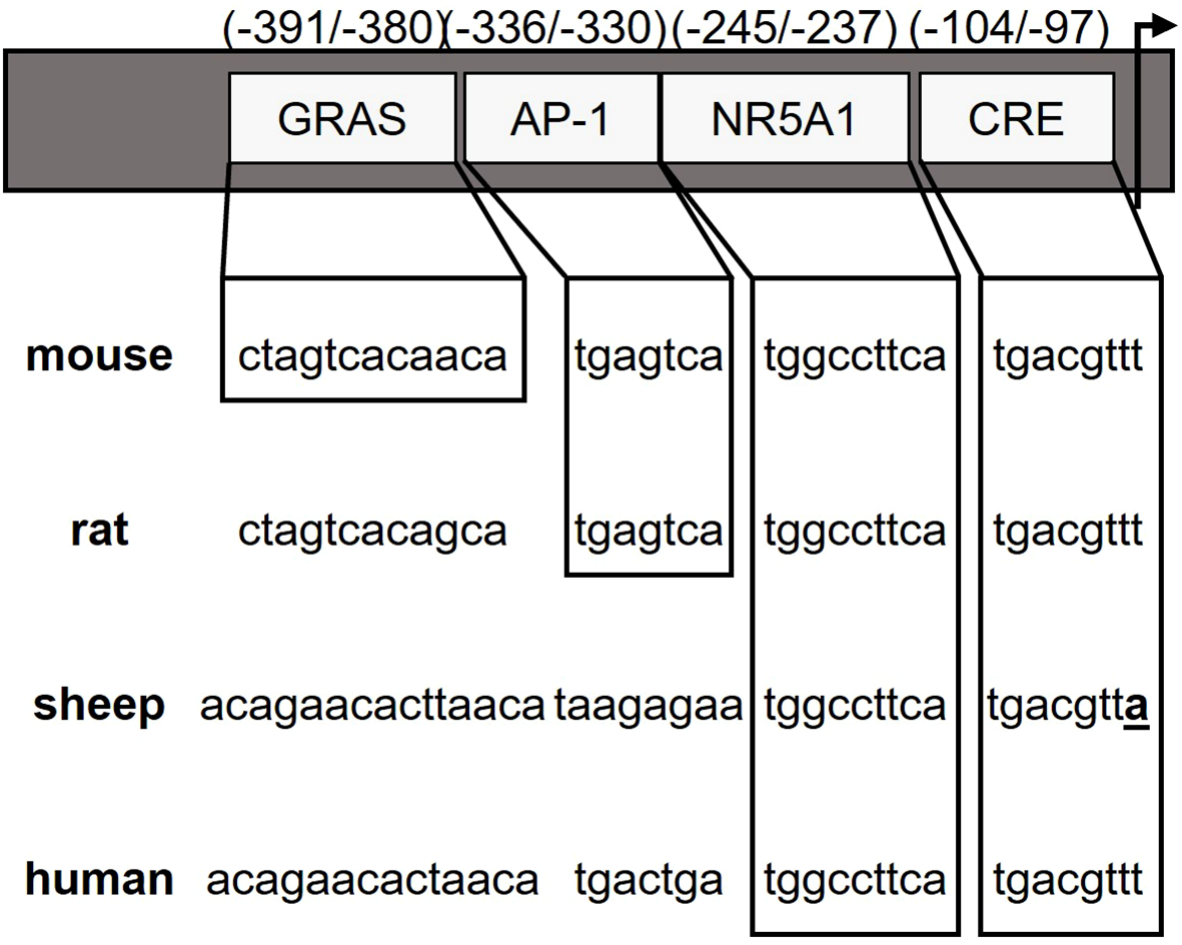
Species differences in GnRHR promoter elements. Promoter regions within -400 bp of the GnRHR start site (arrow) are indicated with sequence similarity between species presented by boxes for specific cis elements and divergence of the ovine CRE element is highlighted.

Both *in vivo* (11, 12) and *in vitro* models (2, 13) support the homologous regulation of *GnRHR* by GnRH. The ability to recapitulate GnRH regulation of the *GnRHR* expression in immortalized gonadotrope cell lines allowed for relatively rapid progress in characterizing the underlying molecular mechanisms. Study of the proximal *GnRHR* promoter has identified several *cis-*regulatory elements; the presence of these elements, their ability to regulate basal *GnRHR* expression and facilitate agonist-induced gene expression, varies by species (summarized in **Figure 1**). In the murine *GnRHR* (*mGnRHR*) promoter these *cis* regulatory elements include the Sequence Underlying Responsiveness to GnRH-1 (SURG-1) (14) as well as an activating protein-1 (AP-1) element that is known to bind transcription factors in the jun and fos families (15) and is critical for mediating GnRH input via MAP kinase signaling. The selective elimination of ERK1 and ERK2 in murine gonadotropes underlies the importance of GnRH induced JNK activation of the AP-1 site for maintaining *mGnRHR* and *Fshb* transcript levels (16). The complexity of AP-1 activation of the *mGnRHR* promoter has also been characterized in immortalized gonadotropes (17), where it is partially mediated by ERK-dependent activation (13) and participates in a tripartite enhancer that includes binding sites for nuclear receptor subfamily 5, Group A, Member 1 (NR5A1, previously known as steroidogenic factor-1), as well as an element referred to as the GnRH receptor activating sequence (GRAS) (12, 18). Furthermore, activin responsiveness of the *mGnRHR* promoter requires both GRAS as well as an additional cis-element located 16-bp 3’ of GRAS referred to as the downstream activin regulatory element (DARE) (19).

As a member of the transforming growth factor (TGFβ) family of growth and differentiation factors, activin contributes to *GnRHR* expression by gonadotropes (20–22). The activin binding protein, follistatin, acts as a primary modulator of the biological effects of activin by sequestering activin and preventing activin binding to its cognate receptor (23). However, divergent biological effects of activin are evident depending on species. In mice for example, activin stimulates both *Fshb* and *mGnRHR* expression (18, 19, 24); whereas in cultured ovine pituitary cells, activin selectively increases FSH secretion and decreases the number of GnRHR available for binding, though this effect on GnRHR is negated in the presence of estrogen (25). Much of this may have to do with the specific activin-responsive elements that are present in the *GnRHR* promoters for each species. Neither GRAS nor DARE has yet to be identified in any species other than the mouse, nor does GRAS confer activin responsiveness to the rat *GnRHR* promoter (18). The convergence of GnRH and activin initiating expression of the *mGnRHR* has been demonstrated by a non-consensus AP-1 binding site that overlaps with a putative SMAD-binding element (SBE) (26), but again, these findings are limited to the murine *GnRHR* promoter.

The consistent upregulation of *GnRHR* expression by GnRH is not at issue. It is the need to deconstruct the species-specific divergence of activin and estrogen regulation of *GnRHR* expression that has resulted in our use of transgenic mouse models to parse whether the *in vivo* findings are the result of transcriptional factors available in the gonadotrope cell, a matter of *GnRHR* promoter regulation, or some combination thereof. Herein we demonstrate that divergence in activin regulation is a result of species-specific differences in the promoter for *GnRHR*.

In many species, including humans and sheep, the increase in estradiol (E_2_) secreted by the pre-ovulatory follicle is obligatory to initiate an increase in GnRH secretion from the hypothalamus and the presence of GnRHR on the surface of the gonadotrope prior to the LH surge (27–31). However, a canonical estradiol response element (ERE) has not been identified in any *GnRHR* gene reported to date (32–36) and the identity of the transcriptional machinery by which E_2_ regulates *GnRHR* expression remains to be determined. Interestingly, a cyclic AMP response element (CRE) in the proximal promoter of *GnRHR* is highly conserved in all species except for the horse (37). Using an adenovirally delivered dominant-negative form of the CRE binding protein (ACREB), we have blocked E_2_ activation of *GnRHR* expression in primary cultures of ovine pituitary cell (38). Herein, we confine our studies of E_2_ regulation to the ovine *GnRHR* (*oGnRHR*) promoter as we have been unable to detect E_2_ regulation of the *mGnRHR* (39). With the use of a transgenic mouse expressing the *oGnRHR* promoter and ovine pituitary cells, we demonstrate that E_2_ activation of the *oGnRHR* promoter requires an intact CRE, and the interaction of CREB or phosphorylated CREB is independent of a canonical ERE. The overall goal of these studies was to provide additional mechanistic understanding of *GnRHR* regulation and continued evidence for the divergence in *GnRHR* regulation between the mouse and the ewe.

## Methods and materials

### Materials

Dr. Pamela Mellon (University of California San Diego, San Diego, CA) generously provided the αT3–1 cells. Dr. Wylie Vale (Salk Institute, La Jolla, CA) provided the adenoviral follistatin (AdCAFS288) and GFP (AdGFP) expression vectors (39). Amplification and purification of AdCAFS288 and AdGFP were performed as previously described (40). Continuous time-release estradiol-17β pellets (2.5 mg over 21 d, Catalogue #E-121) were obtained from Innovative Research of America (Sarasota, FL). Anti-GnRH serum (hereafter referred to as antiserum, AS) was prepared against keyhole limpet hemocyanin-conjugated GnRH in sheep and has been described (41). Restriction and modifying enzymes were purchased from New England Biolabs, Inc. (Beverly, MA) and were used according to supplier’s specifications.

### Plasmids

Construction of plasmids containing 9,100 and 2,700 bp of proximal promoter regions for the *oGnRHR* fused to a luciferase (-LUC) expression vector (-9100LUC, -2700LUC) has been previously described (11). Plasmid -160LUC was constructed by PCR amplification of -2700LUC using the original antisense primer for -2700LUC (5’-GAGCTCGGCACTTCTGATGTT-3’) and a sense primer directed against the appropriate sequence in the 5’ flanking region (5’-TTAAATACAAAGTATCTCAGG -3’) and ligated into the *Sma*I site of the pGL3 vector (Promega Corp., Madison, WI). Additional -115LUC and -79LUC plasmids were similarly constructed by PCR amplification utilizing the -160pGL3, the GLprimer2 (5’-CTTTATGTTTTTGGCGTCTTCCA-3’, Promega Corp., Madison, WI) and a sense primer for either -115 bp (5’-CCTGTGACGTTACCAGCCA-3’) or -79 bp (5’-ACAGGACTCCAAGTGCAATTACA-3’). The CRE in the *oGnRHR* promoter is located within 160 bp of the transcriptional start site. A mutation of the *oGnRHR* promoter CRE (µCRE) was achieved by using a two-step PCR (42) to place an EcoRI recognition site in the context of the both the -160 and the -2700 oGnRHR-LUC constructs.

### Cell culture, transient transfection, and luciferase assays

All cell cultures were maintained in a humidified atmosphere of 5% CO_2_ at 37°C. The αT3–1 cells were cultured in high-glucose DMEM containing 2 mM glutamine, 5% FBS, 5% horse serum, 100 U/ml penicillin, and 100 μg/ml streptomycin sulfate. These cells were transfected for 18 hours utilizing Lipofectamine™ (Thermo Fisher Scientific, Waltham, MA) with oGnRHR-LUC promoter constructs (-2700LUC, -160LUC, -115LUC, -79LUC) as well as the mutated CRE construct (-160µCRELUC) and promoterless control (LUC) (11, 43). To achieve a robust but not maximal response, cells were then treated for 5 h with 10 μM forskolin in DMSO (Sigma, St. Louis, Mo). At the time of sample collection, the media was aspirated, and the cells were washed twice with ice-cold PBS (pH 7.4). Cells were lysed in the wells by the addition of 200 μl lysis buffer (17). Lysates were collected and cellular debris was removed by centrifugation at 16,000 × *g* for 2 min. Cellular lysates were immediately assayed for luciferase activity by adding 20 µl lysate to 100 μl luciferin substrate (Promega Corp, Madison, WI) and measuring luminescence with a Turner model TD-20E luminometer set for a 5 sec delay and 10 sec integration. β-Galactosidase activity was measured in 50 μl of lysate using the luminescent assay system and substrate (Tropix, Bedford, MA) with the same luminometer set for a 10-sec delay and 5-sec integration following manufacturer’s instructions. Luciferase activity in transfection assays was normalized for transfection efficiency (% induction) by dividing the luciferase activity by β -galactosidase activity.

### Generation and screening of transgenic mice

All animal work described herein was performed under veterinary supervision with approval from the Colorado State University Institutional Animal Care and Use Committee (IACUC) and in accordance with the NIH Animal Care and Use Guidelines. All mice used in these experiments were of the FVB inbred strain (44). Animals were maintained under a 14 h light, 10 h dark cycle, received food and water *ad libitum*, and all experiments were conducted using animals older than 6 weeks of age. Genomic DNA was extracted from tail or ear biopsies and analyzed for the presence of the transgene by slot blot hybridization (45, 46) or PCR amplification employing primers specific to luciferase (45) or the transgene. The generation of transgenic mice harboring either approximately 1,900 bp of proximal promoter from the murine *GnRHR* gene fused to the cDNA encoding for luciferase (mGnRHR-LUC) or 9,100 bp of proximal promoter from the ovine *GnRHR* gene fused to the cDNA encoding for luciferase (oGnRHR-LUC) has been described (11). However, cryopreserved embryos from the oGnRHR-LUC animals were thawed and transferred by The Jackson Laboratory (Bar Harbor, ME) and a single oGnRHR-LUC female was used to re-derive the wild-type (WT) promoter.

To generate animals containing a mutated CRE, a construct with an EcoRI site (µCRE) within the ovine -2700LUC plasmid (µCREoGnRHR-LUC) was digested with SacI and BamHI to release the transgene and purified by electroelution. Transgenic founder animals were generated using standard microinjection by the Transgenic and Gene Targeting Core at University of Colorado Denver-Anschutz Medical Campus (Denver, CO). Genomic DNA was extracted from tail biopsies with a REDExtract-N-Amp Kit (Sigma, St. Louis, MO) and analyzed for the presence of the transgene by PCR amplification using primers to luciferase. Four transgenic founder mice (3 males, A-C; 1 female, D) with the µCREoGnRHR-LUC gene were bred to non-transgenic FVB mice and offspring were genotyped specifically for the μCRE transgene. The female founder (Line D) was euthanized during parturition of her first litter as a result of dystocia.

Extensive tissue screening of gonad-intact males and females for all lines harboring the µCRE were performed to confirm tissue-specific expression of luciferase activity as compared to the animals harboring the WT promoter. Luciferase activity was measured from samples of the pituitary gland, brain, gonad, liver, kidney, lung, heart, and spleen.

### Luciferase assays of tissue samples

Luciferase assays were performed as previously described (11). Briefly, tissue samples were prepared by homogenization in 200 μl cold lysis buffer (25 mm glycyl-glycine, pH 7.8; 1.0% Triton X-100, 10 mm MgSO_4_, and 1.0 mm dithiothreitol). Cellular debris was pelleted by microcentrifugation at 16,000 × *g* for 5 min at 4° C. Cellular lysates were immediately assayed for luciferase activity as above. Total protein was precipitated from lysates with 10% trichloroacetic acid and then dissolved in 0.1 n NaOH. Protein concentrations were determined using the bicinchoninic acid (BCA) Assay (Pierce Chemical Co., Rockford, IL), and luciferase activity was adjusted for protein content by dividing the arbitrary light units by the protein content in mg.

### Radioimmunoassays

Concentrations for FSH were determined via radioimmunoassay (47). The reference standards NIH-rFSH-RP2, the antibody anti-rat FSH A621 (1:32,000), and iodinated rat FSH (^125^I-rFSH) were used. Intra-assay coefficient of variation ranged between 4.2 and 8.8%. The inter-assay coefficient of variation was 15.4%. The mean (± SEM) limit of detection for FSH was 43.3 ± 14.2 pg/200 μL.

### Animal treatments

Experiment 1: AdCAFS288 and AdGFP infection of non-transgenic mice.

Male and female non-transgenic mice were gonadectomized. One week post-surgery, mice received a single intraperitoneal (IP) injection of adenovirus expressing either follistatin (AdCAFS288) or green fluorescent protein (AdGFP). Mice were randomly assigned to one of two doses of each virus; 3.9x10^10^ pfu/ml (AdGFP n=5; AdCAFS288 n=4) or 3.9x10^11^ pfu/ml (AdGFP n=5; AdCAFS288 n=3) in 200 μL of phosphate buffered saline (PBS). Three days following injection, trunk blood was collected and serum concentrations of FSH were determined by radioimmunoassay.

### AdCAFS288 and AdGFP infection of mGNRHR-LUC and oGNRHR-LUC transgenic mice

Male and female mGnRHR-LUC (17) and oGnRHR-LUC (11) transgenic mice were gonadectomized. After 7 days, the mice received a single IP injection of 3.9x10^11^ pfu/ml of either AdGFP (mGnRHR-LUC n=8; oGnRHR-LUC n=7) or AdCAFS288 (mGnRHR-LUC n=8; oGnRHR-LUC n=6) in 200 μL of PBS. Three days post-injection, animals were sacrificed, and pituitary glands and livers were harvested for luciferase assay as described. Trunk blood samples were also collected for the determination of serum concentrations of FSH.

### E2 Responsiveness of the oGnRHR Promoter *in vivo*

Extensive validation of the new µCREoGnRHR-LUC animals included luciferase assays from tissues pituitary (Pit), brain (B) gonad (G), liver (Lv), kidney (K), lung (Lg), heart (H), and spleen (S) obtained from transgenic male and female animals from each of the µCRE founder lines (µA n=12 male, n=8 female; µB n=8 male, n=14 female; µC n=13 male, n=5 female). For each founder line, animals that were PCR negative for the transgene were also evaluated (µA n=14 male, n=14 female; µB n=8 male, n=11 female; µC n=16 male, n=10 female). Average luciferase expression in non-transgenic animals (average + 2 standard deviations) was used to determine transgene expression for each of the tissues. Luciferase expression in transgenic (n=21) as compared to non-transgenic (n=19) WT oGnRHR-LUC animals was confirmed before initiating the E_2_ experiments.

To evaluate E_2_ responsiveness of the *oGnRHR* promoter, 7 days after OVX, transgenic females harboring the WT or µCREoGnRHR-LUC transgene were randomly assigned to one of three treatment groups: 1) no treatment (WT n=5; µA n=4; µB n=8; µC n=8); 2) AS but no E_2_ (WT n=8, µA n=7, µB n=10, µC n=8); and 3) AS and E_2_ implant (WT n=6; µA n=7; µB n=7; µC n=8). Those implanted with the 2.5 mg E_2_ pellet were housed separately from the sham (non-E_2_) OVX females. Five days later, females (± E_2_) were given GnRH antiserum (300 μl IP), and at 36–48 hr, tissues (pituitary gland, brain, liver) were harvested and assayed for luciferase activity.

### Electrophoretic mobility shift assays

Ovine pituitary glands (n=2) were obtained from female sheep (ages 3–7 years) immediately following euthanasia and dissociation of ovine pituitary as previously described (48). Cells were treated with 10 nM E_2_ or an equivalent volume of vehicle (ethanol) in media for 30 min (38) before proceeding with nuclear extraction. Cell nuclear extracts were prepared using the Nuclear Extraction Kit (Signosis, Inc., Santa Clara, CA), and EMSAs were performed using the LightShift Chemiluminescent EMSA Kit (Thermo Fisher Scientific, Waltham, MA). Nuclear extracts were incubated at room temperature in 1x Binding buffer from Light Shift kit, 100 mM KCl, 5% (vol/vol) glycerol, 50 ng/μL poly(dI-dC), and 1 mM EDTA, pH 7.5 with unlabeled and biotinylated (probe) oligonucleotides. After incubation at room temperature, the samples were resolved on a 5% nondenaturing polyacrylamide gel prepared in 89 mM Tris-borate and 2 mM EDTA (TBE) buffer, pH 8.3. The specimens were electrotransferred onto a 0.45 μm Biodyne B nylon membrane (Thermo Fisher Scientific, Waltham, MA) at 380 mA for 30 min at 4°C, and crosslinked to the membrane using a CL-1000 UV Translinker and developed using a Chemiluminescent Nucleic Acid Detection Module Kit from Thermo Fisher Scientific (Waltham, MA). Biotinylated probe and unlabeled oligonucleotides (**Table 1**) were custom synthesized by Integrated DNA Technologies, Inc. (Coralville, Iowa). Goat polyclonal p-CREB-1 (Ser 133) (sc-7978) antibody and mouse monoclonal CREB-1 antibody (sc-240X) were purchased from Santa-Cruz Biotechnology (Dallas, TX).

**Table 1:** Oligonucleotides for EMSA.

ovine CRE (ovCRE)	5’-ATAAACCTGTGACGTTACCAGCCAAAG-3’
human CRE (huCRE)	5’-TCTAAACCTGTGACGTTTCCATCTAAAG-3’
consensus CRE (conCRE)	5’-AGAGATTGCCTGACGTCAGAGAGCTAG-3’
nonspecific (nsCRE)	5’-TTTTGTATCTGTCTAGTCACAACAGTTTTT-3’

### Statistical analysis

**Figure 2:**
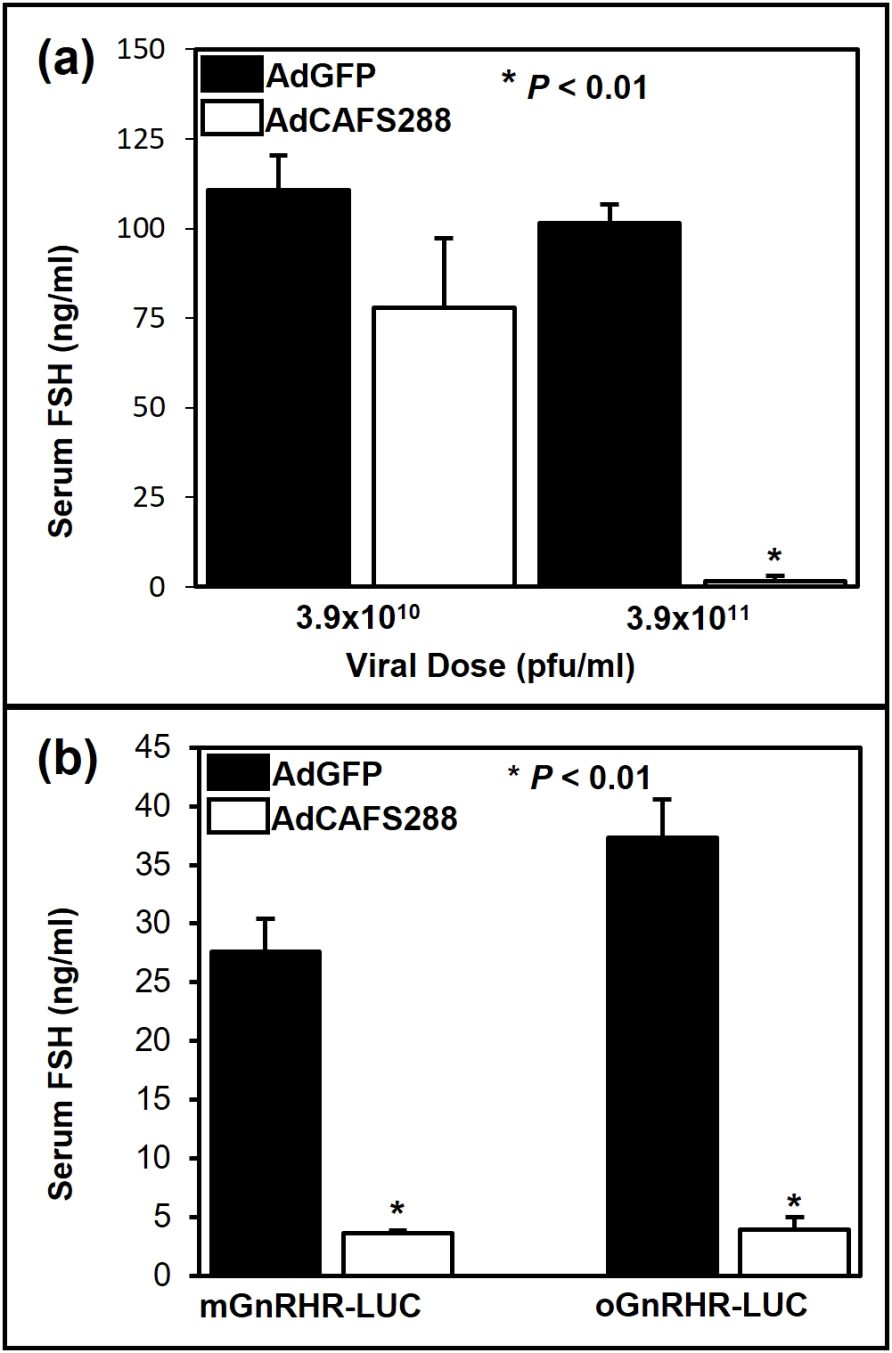
Adenoviral mediated over-expression of follistatin suppresses serum concentrations of FSH. **(a)** Non-transgenic FVB mice were gonadectomized and 7 d later infected with one of two doses of either AdGFP or AdCAFS288. At 3 d post-infection, trunk blood was collected and serum concentrations of FSH were determined by RIA. Values represent the mean + SEM, * (P<0.01) between AdGFP and AdCAFS288 infected mice for each dose. **(b)** Transgenic mice expressing either the wild-type mouse or sheep GNRHR promoters fused to luciferase were gonadectomized and infected as before with a single injection of 3.9 x 10^11^ pfu/ml of either AdGFP or AdCAFS288. At 3 d post-infection, trunk blood was collected and serum concentrations of FSH were determined by RIA. Values represent the mean + SEM. * (P<0.01) between AdGFP and AdCAFS288 infected animals for each line of transgenic mice.

**Figure 3:**
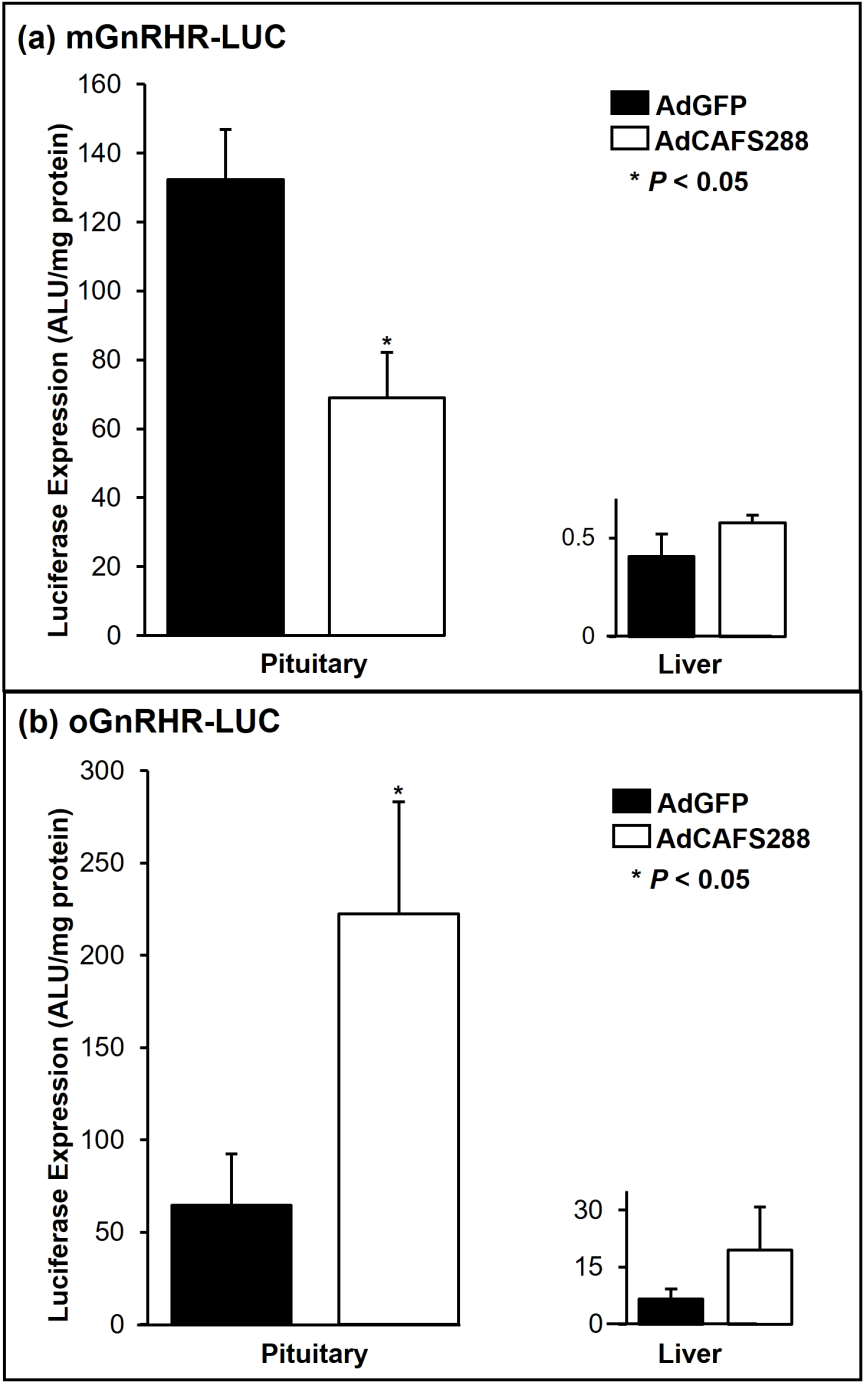
Divergence in follistatin mediated pituitary luciferase expression between the mouse and sheep GnRHR promoters. Transgenic mice expressing either the wild-type mouse **(a)** or sheep **(b)** promoters fused to luciferase were gonadectomized and 7 d later were infected with a single IP injection of 3.9 x 1011 pfu/ml of either AdGFP or AdCAFS288. At 3 d post-infection, the indicated tissues were harvested and assayed for luciferase activity. Luciferase values were adjusted for protein content and are expressed as arbitrary light units (ALU) per mg of protein. Values represent the mean + SEM. * (P<0.05) between AdGFP and AdCAFS288 for each transgenic line of mice.

**Figure 4:**
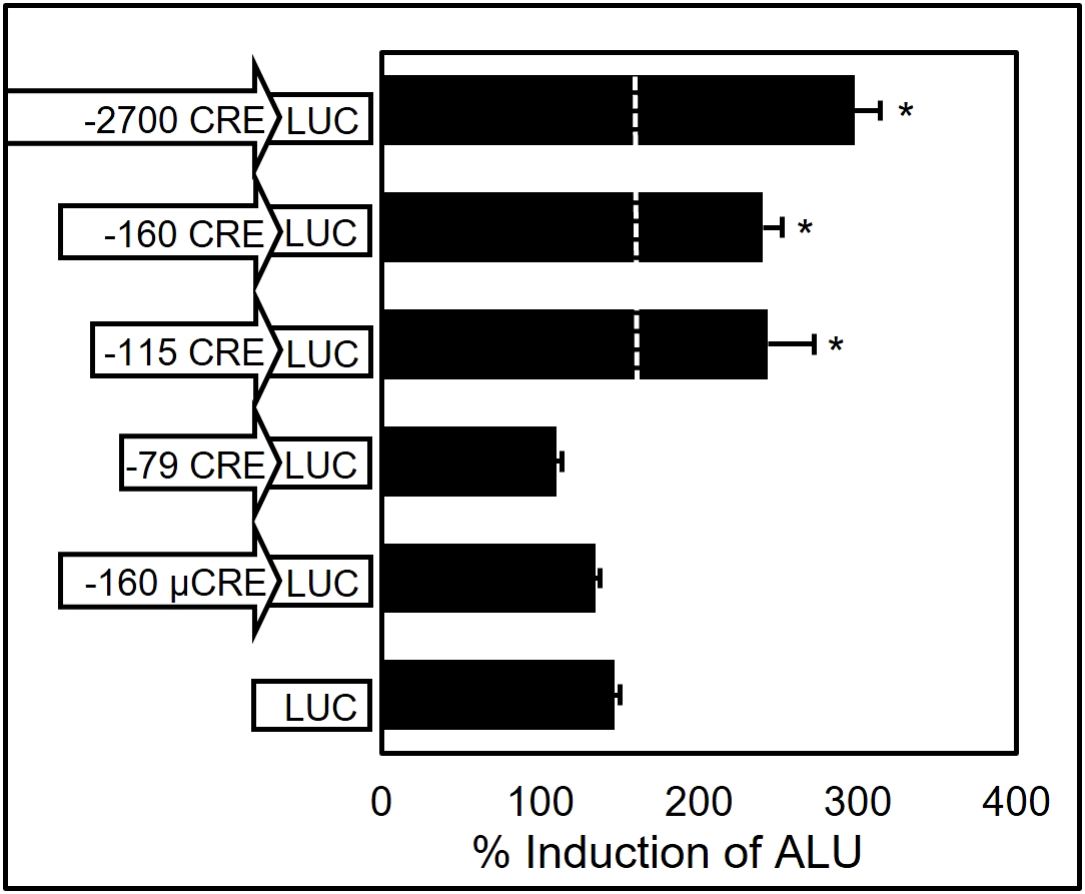
Transient transfection assay of αT3–1 cells with ovine GnRHR gene promoter deletion and mutation constructs reveals that the CRE confers responsiveness of the oGnRHR to forskolin. Cells were transfected for 18 h, then treated for 5 h with 10 μM forskolin in DMSO. Promoter regions with CRE or μCRE are indicated as fused to luciferase (LUC). Values represent mean + SEM percent (%) induction of adjusted luciferase units (ALU) over promoterless (LUC) control. * represents luciferase values significantly different from LUC only (P < 0.05).

Data are expressed as the mean ± the standard error of the mean (SEM). Only when evaluating the µCREoGnRHR-LUC transgene was sex considered a variable in the statistical analysis. For differences in luciferase expression or serum FSH concentration between mice infected with AdGFP versus AdCAFS288, a Student’s T-test was used for analysis. For the experiments involving the µCRE oGnRHR-LUC mice, all data were analyzed using the NCSS8 (Kaysville, UT) statistical software General Linear Model ANOVA, followed by a Tukey-Kramer Multiple Comparison Test. Significance, unless otherwise stated, was *P* < 0.05. Tissues expressing luciferase activity at levels above the mean + 2 Standard Deviations (2SD) of the values in tissues from non-transgenic animals in that population was considered positive for expression of the transgene. Where necessary, data were log transformed to correct for heterogeneity of variance. Means were separated using Student’s T-test and Dunnett’s (**Figures 2**–**4**) or Tukey’s (**Figures 6**, **7**) methods of multiple comparisons.

## Results

### Divergent regulation of ovine and murine GnRHR gene expression by follistatin in transgenic mice

The ability of follistatin to attenuate transcriptional activity of the murine *GnRHR* gene promoter in αT3–1 cells has also been well established (18, 49) and more recently activin has been shown to induce *mGnRHR* transcription *in vivo* (50). Activin has been shown to decrease o*GnRHR* expression in primary ovine pituitary cells (25); however, this effect has never been studied *in vivo*. To address this question, we first tested the effect of increasing viral load on serum concentrations of FSH in non-transgenic mice of the same genetic background (FVB) in which our ovine and murine GnRHR-LUC transgenic lines were constructed. At the 3.9x10^11^ pfu/ml dose, FSH levels were reduced by approximately 90% (*P* < 0.01) in AdCAFS288 infected FVB mice compared to the AdGFP group (**Figure 2a**). Thus, as in rats (40), *in vivo* infection with AdCAFS288 effectively neutralizes activin input to gonadotropes in both male and female wild-type mice Subsequent studies in transgenic mice were conducted using the 3.9 x 10^11^ pfu/ml dose for AdCAFS288 and AdGFP. The adenoviral paradigm described for **Figure 2a** was then applied to mice harboring the transgene consisting of either approximately 1,900 bp of proximal promoter from the murine *GnRHR* gene fused to the cDNA encoding for luciferase (mGnRHR-LUC) or 9,100 bp of the proximal promoter from the ovine *GnRHR* gene fused to the luciferase cDNA (oGnRHR-LUC). The tissue-specific activity of these promoters has been previously established (11, 39). As above, in animals harboring both the ovine and murine *GnRHR* promoters, the AdCAFS288 infection significantly reduced serum FSH levels compared to AdGFP, thus confirming effective neutralization of activin in both transgenic models (**Figure 2b**). However, divergence in follistatin regulation of *GnRHR* expression was observed in pituitary expression of luciferase between the mouse and sheep *GnRHR* promoters (**Figure 3**). Pituitary luciferase expression in the mGnRHR-LUC animals was reduced by approximately 40% (*P* < 0.01) in animals receiving AdCAFS288 as compared to the AdGFP treatment group (**Figure 3a**). In alignment with what has been shown in primary ovine pituitary cells (25), pituitary luciferase expression in the oGnRHR-LUC animals was more than doubled (*P* < 0.05) in the animals receiving AdCAFS288 as compared to the AdGFP treatment group (**Figure 3b**). Follistatin-mediated reduction in luciferase expression in the mouse is equivalent to that which has been observed *in vitro* (49). The adenoviral constructs likely do not cross the blood-brain barrier, therefore evaluation of luciferase expression in the brain may not be biologically relevant and are therefore not shown. Hepatic expression of luciferase, included as a negative control tissue, was at or below detection limits in animals harboring the mouse promoter, but not different between AdCAFS288 as compared to the AdGFP for the promoter of either species (**Figure 3**). Sex differences were not observed in gonadectomized animals and therefore sex was not a variable included in the analysis.

### The ovine GnRHR CRE confers responsiveness to cAMP and forskolin in αT3–1 cells

Despite the absence of significant basal activity of the ovine 5’ flanking region in the murine pituitary-derived αT3–1 cell line (11), there was still a distinct possibility that element(s) conferring responsiveness to endocrine mediators of *GnRHR* gene expression may be located within the promoter fragment and amenable to detection using transient transfection assays. Transfection of αT3–1 cells with o*GnRHR* promoter deletion vectors (-2700LUC, -160LUC, -115LUC, **Figure 4**) or a positive control vector of 1,500 bp of proximal promoter from the human glycoprotein hormone alpha-subunit gene (51–53), with subsequent treatment of the transfected cells with forskolin, led to robust induction (*P* < 0.05) of the *oGnRHR* promoter constructs. These data suggested that the presence of one or more elements located within 160 bp of proximal promoter was responding to cAMP. In fact, examination of this region had revealed the presence of a near-consensus cAMP response element (CRE; ^5’^TGACGTTA^3’^) (54) located between -111 and -104 relative to the start site of translation. To test the potential role of this element, two additional deletions were constructed that deleted (-79LUC) or mutated (-160μCRELUC) the CRE homolog. Forskolin treatment of αT3–1 cells transfected with ovine -2700, -160, and -115 constructs led to a significant increase in luciferase expression that was lost upon deletion or mutation (**Figure 4**).

### Transgenic mice expressing the -9100 μCRE oGnRHR-LUC mice retain tissue specific luciferase expression

**Figure 5:**
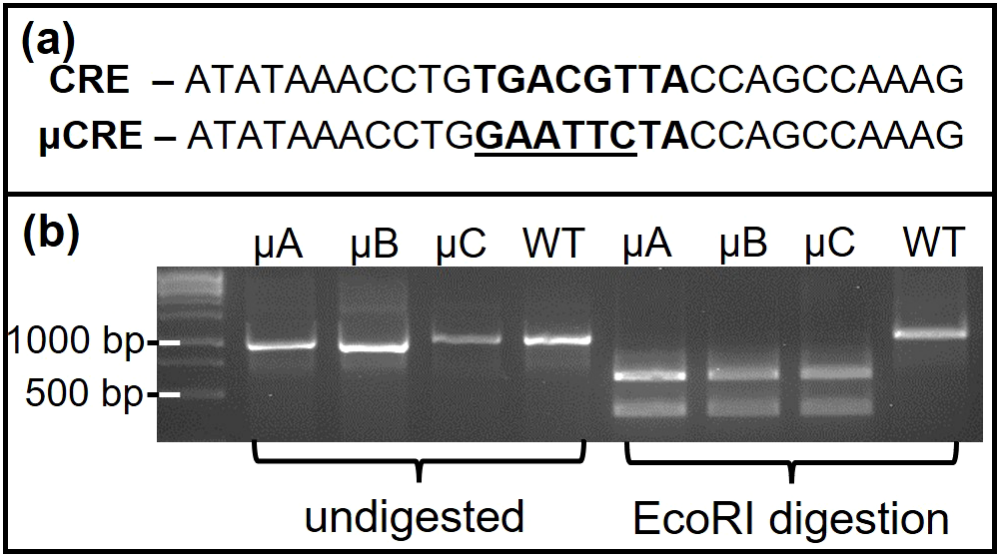
Mutation of CRE is present in µCRE oGnRHR-LUC founders but not WT oGnRHR-LUC animals. **(a)** The (5’-3’) conserved CRE site (TGACGTTA) and the EcoRI restriction site (GATTCC) introduced in -104/-97 bases of oGnRHR-LUC proximal to LUC. **(b)** PCR amplicon of genomic DNA spanning the oGnRHR promoter and LUC gene was digested with EcoRI to reveal the mutation in μCREoGnRHR-LUC founders A-C, but was not present in WT animals.

**Figure 6:**
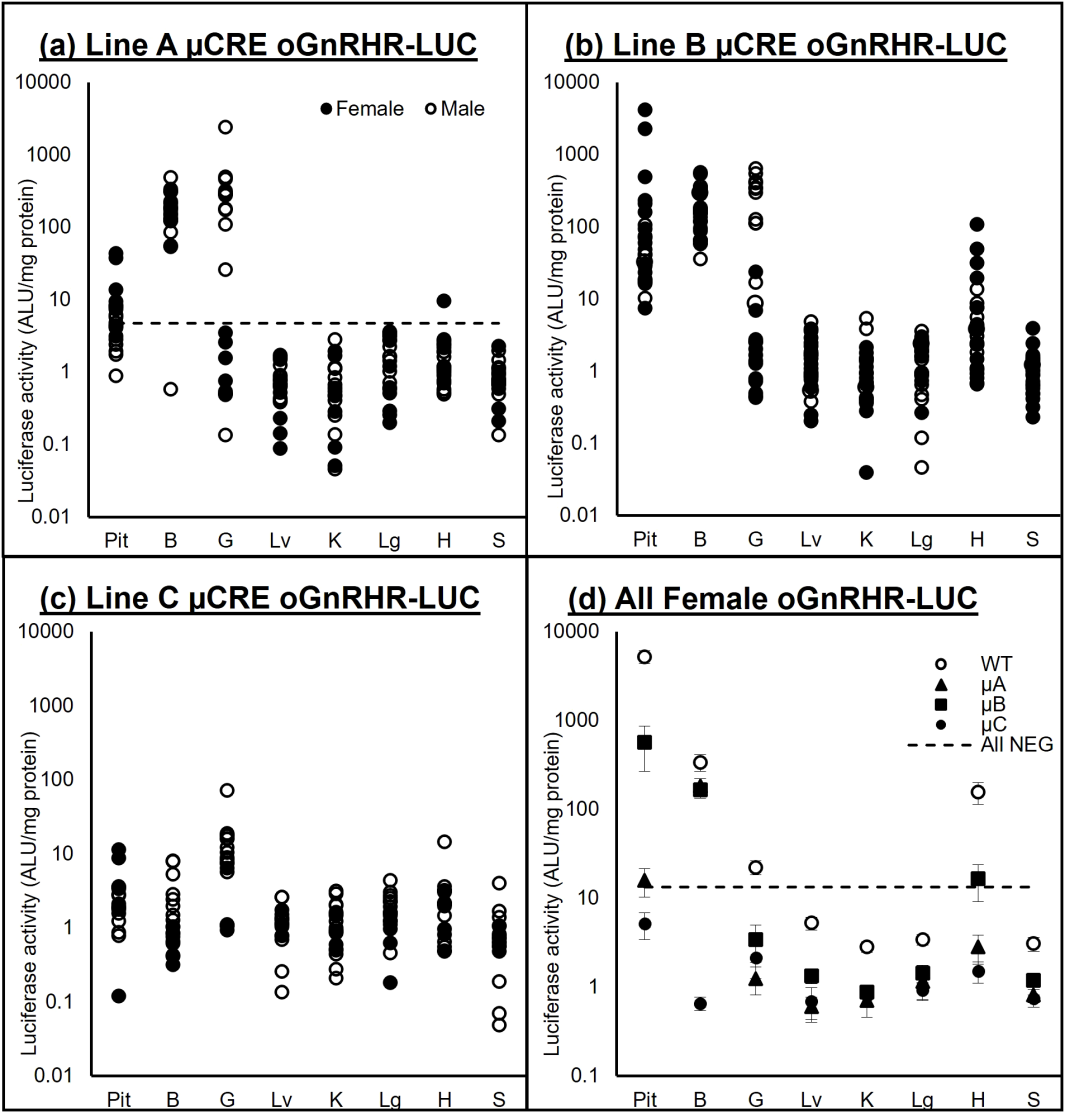
Luciferase activity (ALU/mg protein) in oGnRHR-LUC mice. **(a–c)** Samples from pituitary (Pit), brain (B) gonad (G), liver (Lv), kidney (K), lung (Lg), heart (H), and spleen (S) from males (○) and females (●) of Lines A-C of the µCREoGnRHR-LUC line of transgenic mice. **(d)** Mean (± SEM) tissue luciferase activity of transgenic females from each line (○WT, ▲µA, ■µB, ●µC) of oGnRHR-LUC. Tissue luciferase expression values from negative females from all 4 transgenic lines was averaged + 2 SD to determine the All NEG. Tissue luciferase activity in the μCRE animals was consistent with that of the WT animals, though the amplitude of expression was significantly lower in the μCRE lines. In all lines, luciferase activity was significantly higher in the pituitary gland from transgenic females, the gonad from transgenic males, and in the hypothalamic region of the brain of both transgenic males and females.

Amplification of DNA from the WT and μCRE lines (A-C) of mice, followed by EcoRI restriction enzyme digest of the PCR amplicon, confirmed the presence of the mutated CRE in the -9100 μCRE oGnRHR-LUC A-C lines of mice (**Figure 5**). To confirm tissue-specific luciferase expression in these animals, extensive tissue screens of luciferase-positive and negative animals were performed (**Figures 6a–c**). A confidence interval was constructed using the mean + 2SD from tissues collected from animals that were PCR-negative for the luciferase transgene from all 4 transgenic lines. Tissue luciferase activity in the μCRE animals was consistent with that of the WT animals, though the amplitude of expression was significantly lower in the μCRE lines (**Figure 6d**). In all lines, luciferase activity was significantly higher in the pituitary gland from transgenic females, the gonad from transgenic males, and in the hypothalamic region of the brain of both transgenic males and females; whereas the liver was consistently among the lowest luciferase-expressing samples. Interestingly, both transgenic Line B and WT females had increased luciferase activity in the heart.

### A functional CRE is necessary to confer estradiol responsiveness to the oGnRHR promoter

**Figure 7:**
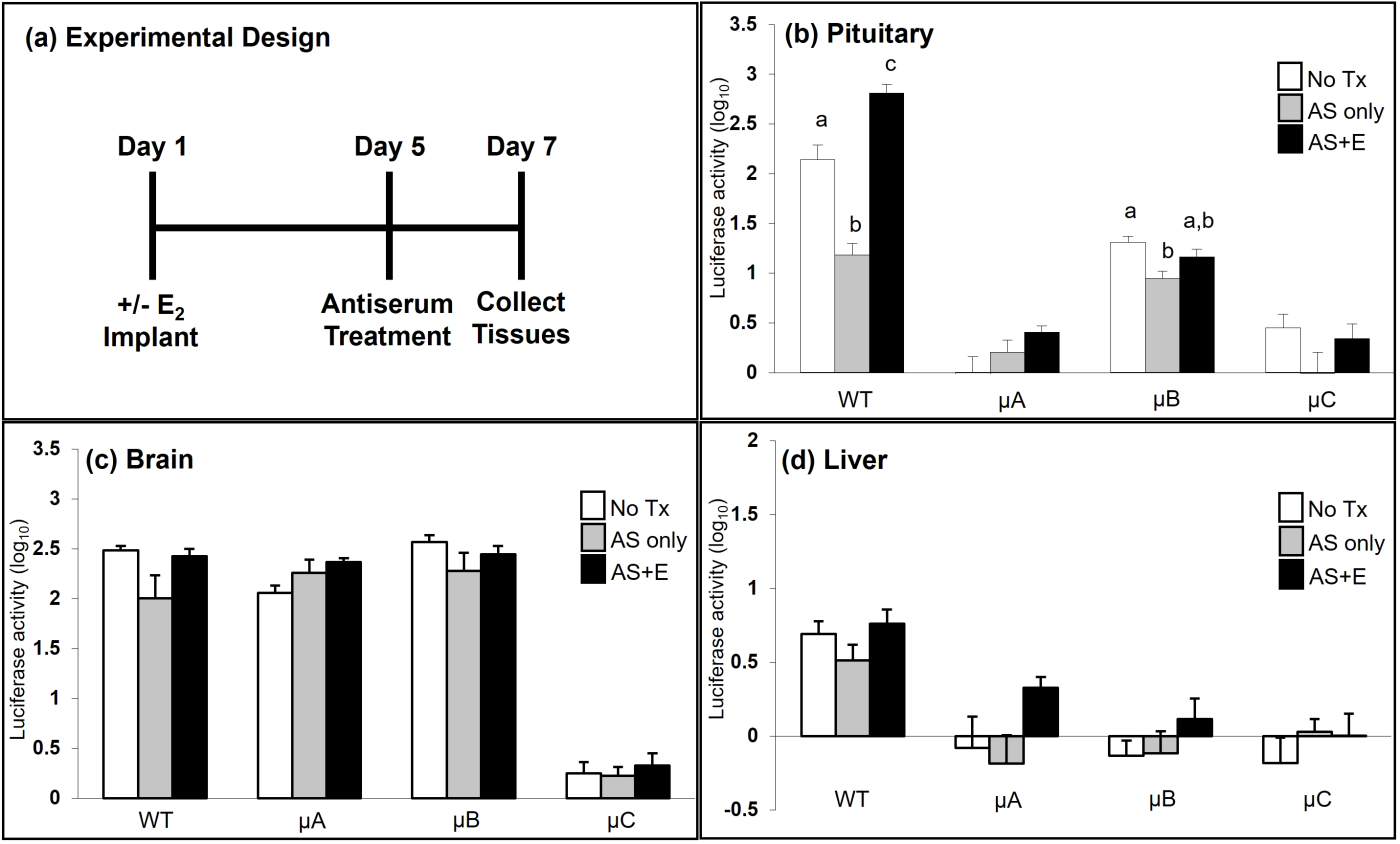
A functional CRE is necessary to confer estradiol responsiveness to the *oGnRHR* promoter. **(a)** Seven days after OVX, females (WT, and A-C of the µCRE -9100 oGnRHR-Luc) were randomly implanted with a 2.5 mg E_2_ pellet (Day 1) and housed separately from non-E_2_ (sham procedure) OVX females. Five days later (Day 5), females (± E_2_) were given GnRH antiserum (AS, 300 μl IP) or sham injection, and at 36–48 hr (Day 7) tissues were harvested for luciferase activity luciferase activity (ALU/mg protein). Data were log transformed and expression in the pituitary gland **(b)**, brain **(c)**, and liver **(d)** are presented. ^a,b,c^ indicate differences between treatment, within the WT or transgenic line for the tissue indicated, *P* < 0.05.

GnRH regulation of pituitary *GnRHR* expression was observed in transgenic mice with both the WT and µCRE versions of the *oGnRHR* promoter following treatment with GnRH AS. Specifically, in both the WT promoter and µCRE line B animals, blockade of GnRH with AS resulted in a significant decrease in pituitary luciferase expression (**Figure 7b**). In the same groups of animals, when compared with no treatment, treatment with AS and E_2_ resulted in a significant increase in pituitary luciferase expression in the animals with the WT *oGnRHR* promoter, but not in the µCRE line B *oGnRHR* promoter animals. While the changes in pituitary luciferase expression for the µCRE lines A and C following treatment with AS and/or E_2_ were not significantly different from the untreated animals, similar trends were observed (**Figure 7b**). There was no evidence of GnRH or E_2_ regulation of brain (**Figure 7c**) or liver (**Figure 7d**) luciferase expression in either the WT or µCRE *oGnRHR* promoter animals.

**Figure 8:**
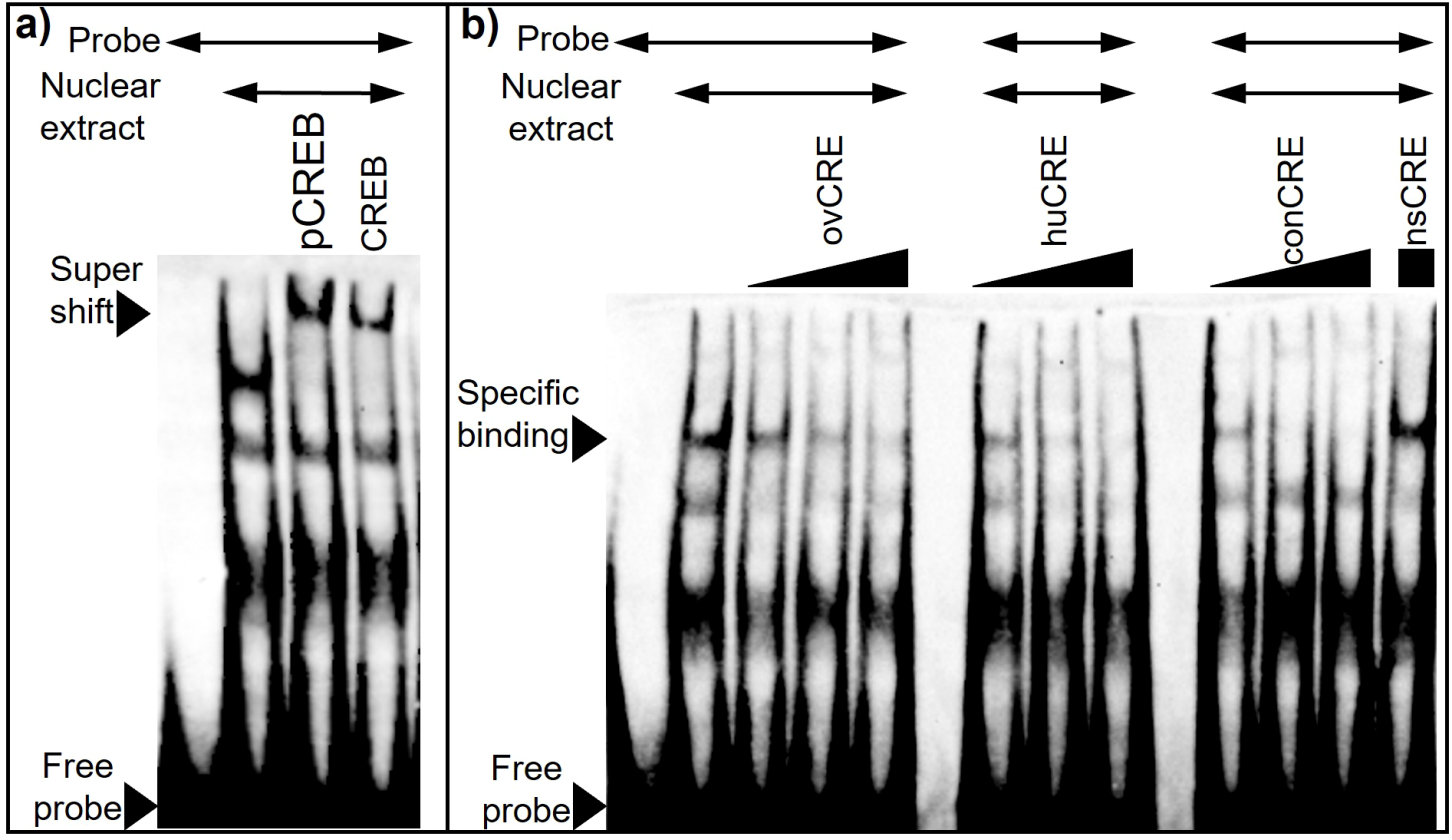
CREB binding is detected at the ovine CRE. **(a)** Both CREB and phosphorylated CREB (pCREB) are associated with DNA binding elements in ovine nuclear extract. Nuclear extract (5 ug) from ovine pituitary cells was incubated for 15 min with 1 µg of antibody for either CREB or pCREB, then incubated an additional 20 min with biotinylated oCRE oligonucelotide. **(b)** Human and consensus CRE elements compete for DNA binding elements in ovine pituitary nuclear extract. Nuclear extract (10 ug) was incubated for 20 min with biotinylated oCRE oligo. Specificity of DNA-protein interactions was assessed by competition with unlabeled oligonucleotides for ovine (ov), human (hu), and consensus (con) CRE at 10, 50, and 100 molar excess, and nonspecific (ns) CRE at 100 molar excess.

*CREB binding is detected at the ovine CRE.* While the transgenic mouse work indicates that a functional CRE binding domain of the ovine proximal *GnRHR* promoter is necessary to confer E_2_ input to the *oGnRHR*, the identity of the transcriptional machinery that mediates E_2_ input to the receptor in the ovine pituitary remains unknown. No canonical ERE is located within the 9,100 bases of the proximal *oGnRHR* promoter. EMSA using nuclear extract from ovine pituitary cells demonstrated competition of the biotinylated ovine CRE with increasing concentrations of ovine, human, and the consensus CRE oligonucleotide sequences, but not the non-specific CRE oligonucleotides (**Figure 8a**). Thus, CRE binding is specific with equivalent affinity across CRE oligonucelotide sequences. Further evaluation demonstrated that both CREB and phosphorylated-CREB (pCREB) are associated with elements in ovine nuclear extract, as demonstrated by the supershift that occurs with or without the presence of additional unlabeled oligonucelotides (**Figure 8b**). However, exposure of the ovine pituitary cells to E_2_ failed to result in any additional interactions or gel-shifts (data not shown). Thus, the identity of the element that confers E_2_ signaling to the ovine *GnRHR* remains unknown.

## Discussion

The divergent mechanisms that regulate the female hypothalamic-pituitary-ovarian (HPO) axis demonstrate that there is no single best model or species. In the animal models predominantly used to study the HPO, GnRH input to the gonadotrope is unequivocally the most important and consistently positive endocrine regulator of *GnRHR* expression (55–61). However, the effect that the pulsatile nature of GnRH signaling has on the GnRHR and subsequent signaling pathways and transcriptional mechanisms that result in gonadotropin synthesis and secretion (62) confound the inherent effect of GnRH on *GnRHR* expression when gonadotropins or ovulation are used as the measurable factor. The initial murine-derived immortalized cell lines (i.e. αT3–1 cells, LβT-2) (63–65) are inherently valuable and have permitted innumerable experiments to study gonadotropes function and signaling. The more recently developed mPitA-12/3 cell line is presumed to be from a fully differentiated cell type and responds to GnRH, but not E_2_, treatment with an increase in *GnRHR* expression (66).

At issue is not the value of the model, but the diversity of the results gained from the different model systems. For example, myostatin as a regulator for FSH in rodents has been described, but the conservation of this mechanism in non-rodent species has yet to be determined (67). The study of activin and follistatin on mouse and rat *GnRHR* expression using immortalized cell lines and/or transgenic mice has consistently demonstrated distinct mechanisms (18, 50). While elements of these mechanisms, including FOXL2 (50) and AP-1 (43) sites are found in the human *FSHB* (68) and *GnRHR* (69) promoter, the GRAS (43) and DARE sequences found in rodents are not conserved in either the ovine or human *GnRHR* promoter (70). It is therefore not surprising that while follistatin overexpression is able to decrease serum FSH in transgenic mice expressing either the mouse or the sheep *GnRHR* fused to luciferase, we have demonstrated divergence in luciferase expression between the mouse and ovine *GnRHR* promoters (**Figure 3**). These findings are also consistent with treatment of ovine pituitary cells with either inhibin or activin-A (4, 25, 71).

*Estradiol regulates* oGnRHR *expression through unresolved mechanisms.* Work in mice (39) and sheep (11) consistently demonstrates that GnRH input to the *GnRHR* promoter is necessary to confer basal expression of *GnRHR*, but does not eliminate the input of E_2_ in the upregulation of luciferase expression in the *oGNRHR* promoter (11). The use of antiserum directed against GnRH effectively neutralizes GnRH input to the *GnRHR* promoter while not disrupting receptor expression. The indisputable ability of estradiol to enhance ovine pituitary sensitivity by increasing the number of GnRHR *in vivo* and *in vitro* (30, 58, 72–74) resulted in the initial use of transgenic mice expressing o*GnRHR* promoter constructs fused to luciferase expression vectors. These animals revealed E_2_ regulation of the o*GnRHR* that could not be elicited in gonadotrope-derived cell lines (11); although the LβT-2 cell demonstrates a moderate increase in *GnRHR* expression following E_2_ treatment (75), the αT3–1 cells actually demonstrate a decrease in GnRHR numbers and mRNA following E_2_ treatment (76, 77). The female rat also appears to be responsive to E_2_ *in vivo* (78, 79), but the changes in GnRHR number are most likely attributable to changes in GnRH secretion (3). Furthermore, GnRH (independent of E_2_) is able to activate an ERE driven luciferase reporter and transcriptional activation of the immediate early response gene *fosB* via an interaction of ERα with the histone acetyltransferase p300/CREB-binding protein-associated factor (PCAF) (80). While the *fosB* promoter contains an ERE, there is no canonical estradiol response element (ERE) in the rat, mouse, or sheep *GnRHR* promoter (36, 70, 81).

*The data reported herein demonstrate that CRE in the oGnRHR promoter is necessary but not sufficient to mediate E_2_ input to the oGnRHR promoter.* Unlike many other transcriptional factors, the CRE is conserved in the proximal region of the sheep, mouse, human, and rat *GnRHR* promoters (70). The *oGnRHR* promoter is responsive to forskolin in αT3–1 cells, but this responsiveness is lost with mutation of the CRE. The first indication of the importance of the CRE for conferring the E_2_ signal to the ovine *oGnRHR* came from adenoviral delivery of a dominant negative form of CREB (ACREB) eliminating the predicted E_2_ mediated increase in GnRHR number (38). In the reported EMSAs, there was no supershift observed in the presence of E_2_, but this is not conclusive evidence that CREB and E_2_ are not interacting. Converging mechanisms involving parallel enhanceosome or mediator complexes (82), including those that may be derived from a membrane site of action, may be involved. Treatment of ovariectomized ewes with an IM bolus of E_2,_ or dispersed ovine pituitary cells with membrane-impermeable forms of E_2_, both result in a decrease in LH secretion followed by an increase in GnRHR number (38). However, in ovine pituitary cells, the dominant-negative form of ESR1 (L540Q), a mutation site known for co-activator binding (83, 84), is able to eliminate E_2_ activation of *GnRHR* (38). With mechanisms that clearly reside in different parts of the gonadotrope, it is altogether possible that the method by which these mechanisms converge has yet to be elucidated. This work also suggests that a membrane-associated ESR1 is the likely candidate to mediate E_2_ activation of the *GnRHR* promoter (38).

The µCRE oGnRHR-LUC mouse clearly demonstrates that CRE mutation does not eliminate GnRH input to luciferase expression but does eliminate E_2_ upregulation of luciferase. Therefore, future studies of the interaction of E_2_ with the CRE region of the proximal *GnRHR* promoter will require pituitary-specific elimination of ESR1 (85) in the context of a gonadotrope-specific ESR1 knockout (86) and the oGnRHR-LUC mouse to determine the functional contribution of ESR1 *in vivo*. Alternatively, the use of other technologies to eliminate the signal-to-noise ratio within the ovine pituitary gland (48) may be promising.

## Data Availability

The raw data supporting the conclusions of this article will be made available by the authors, without undue reservation.
